# Patientenrechte im Kontext des Beschwerdemanagements – Die Einsichtnahme in die Behandlungsunterlagen

**DOI:** 10.1007/s00132-021-04108-6

**Published:** 2021-04-20

**Authors:** M. Schwarze, K. Anthes-Stöcking, P. Glanzmann, M. Schiltenwolf

**Affiliations:** 1 Klinik für Orthopädie und Unfallchirurgie, Zentrum für Orthopädie, Unfallchirurgie und Paraplegiologie, Universitätsklinikum Heidelberg, Schlierbacher Landstraße 200a, 69118 Heidelberg, Deutschland; 2 Gutachterkommission Bezirksärztekammer Nordbaden, Karlsruhe, Deutschland

## Einleitung

Nicht selten kommt es im medizinischen Alltag zu Beschwerden von Patienten. Für alle Beteiligten ist dies regelhaft eine unangenehme Situation, mit der es jedoch professionell umzugehen gilt. Hierfür gibt es u. a. vor dem Hintergrund des Patientenrechtegesetzes (PatRG) und weiteren Normen, wie z. B. der (Muster‑)Berufsordnung (MBO), einige Dinge zu berücksichtigen.

## Rechtlicher Hintergrund

Im Jahre 2013 wurde das Gesetz zur Verbesserung der Rechte von Patientinnen und Patienten (Patientenrechtegesetz) verabschiedet. Dieses Gesetz orientiert sich am Leitbild des mündigen Patienten und soll „einen wesentlichen Beitrag zu mehr Transparenz und Rechtssicherheit“ leisten [1]. Änderungen in weiteren Gesetzen wie dem Bürgerlichen Gesetzbuch, dem Sozialgesetzbuch V, dem Krankenhausfinanzierungsgesetz und der Bundesärzteordnung wurden ebenso vorgenommen, wie Neuregelungen auf dem Gebiet des zivilrechtlichen Behandlungs- und Arzthaftungsrechts [1, 2]. Wichtiger Bestandteil des PatRG war die Einführung des „Behandlungsvertrages“ (§§ 630 a–h BGB). Hierbei handelt es sich im Wesentlichen um eine Kodifizierung der höchstrichterlichen Rechtsprechung der vorangegangenen Jahrzehnte [3]. Darüber hinaus regelt es neben den vertragstypischen Pflichten auch anwendbare Vorschriften, Mitwirkung der Vertragsparteien, Informationspflichten, Aufklärungspflichten, die Einwilligung der Patienten sowie die Dokumentation der Behandlung und die Beweislast bei Haftung für Behandlungs- und Aufklärungsfehler. Zudem regelt es das Recht auf Einsichtnahme in die Patientenakte durch den Patienten.

In § 630g Absatz 1 BGB ist hierbei festgelegt:

Dem Patienten ist auf Verlangen unverzüglich Einsicht in die ihn betreffende Patientenakte zu gewähren, soweit der Einsichtnahme nicht erhebliche therapeutische oder sonstige erhebliche Rechte Dritter entgegenstehen. Die Ablehnung der Einsichtnahme ist zu begründen. § 811 ist entsprechend anzuwenden.

Mit dieser Formulierung weicht das BGB als zivilrechtliche Handlungsgrundlage von der MBO (aktuelle Fassung vom 14.12.2018) ab. Diese legt in § 10 Absatz 2 fest:

Ärztinnen und Ärzte haben Patientinnen und Patienten auf deren Verlangen in die sie betreffende Dokumentation Einsicht zu gewähren, soweit der Einsichtnahme nicht erhebliche therapeutische Gründe oder erhebliche Rechte der Ärztin, des Arztes oder Dritter entgegenstehen. Auf Verlangen sind der Patientin oder dem Patienten Kopien der Unterlagen gegen Erstattung der Kosten herauszugeben.

In der Gesetzesbegründung zu § 630g heißt es: „Der Patient hat ein schutzwürdiges Interesse zu wissen, wie mit seiner Gesundheit umgegangen wurde, welche Daten sich dabei ergeben haben und wie die weitere Entwicklung eingeschätzt wird. Die Regelung greift die Rechtsprechung des Bundesverfassungsgerichts aus dem Jahre 2006 (BVerfG NJW 2006, 1116) auf und dient insbesondere der Umsetzung des Rechts des Patienten auf informationelle Selbstbestimmung“ [2].

Entsprechend der Vorschrift ist demnach dem Patienten „unverzüglich“, was „ohne schuldhaftes Verzögern“ und je nach Fallgestaltung zwischen 14 Tage bis 1 Monat bedeutet, die Einsicht zu gewähren [4]. Für die Geltendmachung seines Rechts zur Akteneinsicht muss der Patient keine spezifischen Gründe nennen. Therapeutische Gründe können jedoch argumentativ dazu verwendet werden, einem Patienten die Einsicht partiell oder vollständig zu verwehren (therapeutischer Vorbehalt). Möglich wäre dies, wenn angenommen werden kann, dass der Patient durch die Einsicht einen erheblichen gesundheitlichen Schaden erleidet und/oder wegen derer die Möglichkeit einer Selbstschädigung besteht. In diesen besonderen Einzelfällen ist es erforderlich, dass die zu berücksichtigenden Belange sorgfältig ermittelt und auf konkrete und substantiierte Anhaltspunkte gestützt werden können. Möglicherweise kommt aber auch eine durch den Behandelnden unterstützende oder auch begleitende Einsichtnahme in Betracht; auch könnte eine dritte Person dem Patienten vermittelnd für die Einsichtnahme zur Verfügung gestellt werden. Maßgebend sind die Umstände im Einzelfall [2]. Eine Grenze erreicht das Einsichtsrecht des Patienten jedoch, sofern die Dokumentation ihrerseits schutzwürdige Informationen über die Persönlichkeit Dritter beinhaltet.

Eine Besonderheit der Formulierung im BGB ist, dass die Rechte des Arztes keine besondere Erwähnung finden. Dieser ist nicht als „Dritter“ gefasst. Daher sind auch Inhalte zu persönlichen Eindrücken oder subjektiven Wahrnehmungen des Behandelnden auszuhändigen. Der Gesetzgeber stellt diesbezüglich im Regelfall Rechte des Patienten über die der Behandelnden.

Die Einsichtnahme hat, mit Ausnahme von wichtigen Gründen, an dem Ort zu erfolgen, an dem sich die Unterlagen befinden (Erfüllungsort).

Gemäß § 630g Absatz 2 BGB kann der Patient jedoch auch elektronische Abschriften von der Patientenakte verlangen. Er hat in diesem Falle dem Behandelnden die entstandenen Kosten zu erstatten. Die Abschriften können sowohl von einer in Textform erstellten Dokumentation als auch von elektronischen Dokumenten und gegebenenfalls auch in Form maschinenlesbarer Datenkopien oder Dateien in elektronischer Form angefertigt werden [2]. Zuletzt wurde jedoch durch das LG Dresden mit Urteil vom 29.05.2020 (Aktenzeichen 6 O 76/20) die bisher unstrittige Frage der Kostenübernahme überworfen. Hintergrund war ein Klageverfahren, in welchem die Herausgabe der personenbezogenen Daten unter Verweis auf Art. 15 Abs. 3 der Verordnung (EU) 2016/679 (Datenschutz-Grundverordnung) erfolgte. Im Wortlaut dieses Absatzes ist definiert: „Der Verantwortliche stellt eine Kopie der personenbezogenen Daten, die Gegenstand der Verarbeitung sind, zur Verfügung. Für alle weiteren Kopien, die die betroffene Person beantragt, kann der Verantwortliche ein angemessenes Entgelt auf der Grundlage der Verwaltungskosten verlangen. …“. Da dieser Absatz Kosten für die in Anspruch nehmende Person erst für „weitere“ Kopien vorsieht, war nach Ermessen des Gerichts die Erstauskunft kostenfrei. Das LG Dresden betonte hiermit das Primat des Europarechts.

Über diese rechtliche Grundsteinlegung der Einsichtnahme in die Behandlungsunterlagen hinaus beinhaltet das PatRG weiterhin Änderungen im SGB V. Hier wurde u. a. ein Beschwerdemanagement zur Förderung der Fehlervermeidungskultur in § 135a „Verpflichtung der Leistungserbringer zur Qualitätssicherung“ verankert. § 135a Abs. 2 Nr. 2 SGB V verpflichtet zur Einführung eines Qualitätsmanagements, wozu in Krankenhäusern auch die Durchführung eines patientenorientierten Beschwerdemanagements gehört. Wesentliche Elemente sind hierbei die Unterrichtung des Patienten in geeigneter Form über die Beschwerdemöglichkeiten vor Ort, zügige und transparente Bearbeitung der Beschwerden, Unterrichtung über das Ergebnis und mögliche Konsequenzen sowie transparente Regelungen in Bezug auf die Stellung und die Kompetenzen der mit dem Beschwerdemanagement betrauten Personen [5, 6].

## Kasuistik

Eine 44-jährige Patientin stellte sich für eine klinisch-radiologische Verlaufskontrolle in der allgemeinen Hochschulambulanz einer Universitätsklinik vor. Bekannt war bei der Patientin eine Brustwirbelfraktur, welche sie sich ca. 1 ½ Monate zuvor traumatisch zugezogen hatte. Zusätzlich wurden patientenseitig Schwindel und Armbeschwerden seit dem Unfallereignis beklagt, weswegen extern zusätzlich eine MRT der Halswirbelsäule veranlasst wurde, welches die Patientin zwecks Besprechung zu dem Termin mitbrachte. Eine HNO-ärztliche Abklärung des Schwindels verblieb ohne pathologischen Befund.

Die behandelnde Assistenzärztin führte im Rahmen der Vorstellung eine abschließende Röntgenkontrolle der konservativ behandelten Brustwirbelkörper(BWK)-Fraktur durch. Da die Aufnahme aufgrund von Überlagerungen für die Ärztin schwer zu beurteilen war, zog sie einen erfahrenen Kollegen der Wirbelsäulenabteilung hinzu. Diesem war eine ausreichende Beurteilung des Bildmaterials möglich, sodass initiale Zweifel der jüngeren Kollegin aufgehoben werden konnten. Beide Kollegen versuchten der Patientin die radiologischen Befunde des Röntgenbildes und der MRT-Bilder der Halswirbelsäule laienverständlich zu vermitteln. Dies beinhaltete auch die Aufklärung über den fehlenden Zusammenhang zwischen dem Verschleißbefund der Halswirbelsäule, der aktuell behandelten BWK-Fraktur und der beklagten Schwindelsymptomatik. Bei der Patientin verblieben jedoch, trotz aller Bemühungen der Behandler, Zweifel an der Qualität und der Zweckmäßigkeit der durchgeführten Bildgebung und Unklarheiten bezüglich der radiologischen Befunde. Zudem empfand sie den Umgang der Assistenzärztin als unfreundlich und sah Widersprüche in den Aussagen beider Behandler. Die Patientin formulierte daher eine schriftliche Beschwerde an den Klinikdirektor mit der Bitte um die abschließende Erklärung der radiologischen Befunde. Darüber hinaus wünschte sie eine Kopie der Bildgebung sowie der gesamten digitalen Behandlungsakte, einschließlich der handschriftlichen Aufzeichnungen auf dem Postweg.

Im Rahmen des standardisierten Beschwerdemanagements wurden beide Behandler zu schriftlichen Stellungnahmen aufgefordert. Diese flossen in die abschließende an die Patientin gerichtete Stellungnahme ein. Da im vorliegenden Fall keine Argumente gegen die Aushändigung der Behandlungsunterlagen vorlagen, wurden diese der Patientin überlassen. Die Kosten hierfür verblieben bei ihr.

## Kommentar

Mit der Einführung des PatRG im Jahre 2013 wurden die Patientenrechte mit dem Ziel der Rechtssicherheit und Transparenz verabschiedet. Der neu definierte Behandlungsvertrag regelt hierbei u. a. Möglichkeiten und Grenzen des Patienten, auf die personenbezogene Dokumentation zuzugreifen. Auch nunmehr 8 Jahre nach Einführung sind jedoch der Mehrheit der Patienten ihr Rechte kaum bekannt. Hart bezog sich in seiner Publikation aus dem Jahre 2019 auf zum Teil eigene Untersuchungen und konstatierte, dass 60 % der Patienten und 32 % der Ärzte über keine spezifischen Kenntnisse des PatRG verfügten. Wenngleich den Ärzten mehrheitlich zwar das PatRG selber nicht bekannt war, so waren es zumindest seine wesentlichen Rechte. Patienten hingegen suchten überwiegend nicht nach spezifischen Informationen zu diesem Thema und waren überwiegend mit der Behandlung zufrieden. Sie beklagten jedoch insbesondere das Informations- und Aufklärungsverhalten der Behandler und Leistungsträger [3]. Die Zahlen verdeutlichen jedoch, dass trotz Einführung des PatRG keine durchgreifende Verbesserung der patientenseitigen Kenntnisse eingetreten ist. Bereits 2010 veröffentlichte die Bertelsmann Stiftung im „Gesundheitsmonitor 2010“ Zahlen von Befragungen. Hierbei konnten gerade einmal knapp 39 % der Befragten (1789 Bürgerinnen und Bürger im Alter von 18–79 Jahren) alle ihnen gestellten Fragen zu Patientenrechten richtig beantworten [7].

Wie sollte aber nun gehandelt werden, wenn das eigene Handeln, wie in der Kasuistik exemplarisch dargestellt, derart kritisch hinterfragt wird und der Patient von seinen Rechten Gebrauch macht? Götz bezeichnete Beschwerden als öffentlich geäußerte Feststellungen einer enttäuschten Erwartung. Sie stellen die subjektive Sicht des Patienten dar und sollten vom Betroffenen nicht persönlich genommen werden [8]. Aus Sicht des Patienten ist es zu einer Diskrepanz zwischen Bedürfnissen, Wünschen und Erwartungen über ihre eigene Behandlung und der tatsächlichen Umsetzung gekommen. Patienten benötigen eine Möglichkeit, dies auszudrücken. Diese ist rechtlich verankert im patientenorientierten Beschwerdemanagement. Management ist hierbei definiert über alle unternehmerischen und betrieblichen Steuerungs- und Koordinierungsaufgaben und verfolgt das Ziel der Qualitätssicherung [9]. Neben verschiedenen Dienstleistungen und Maßnahmen in der Patientenorientierung spielen auch menschliche Qualitäten wie Empathie, Kompetenz, Freundlichkeit und Zuverlässigkeit eine bedeutsame Rolle [10]. In einem modernen Qualitätsmanagement ist dies ebenso Bestandteil wie die Implementierung von Maßnahmen in der Patientenorientierung. Wesentlicher Bestandteil ist hier das Erkennen von Erwartungen und Bedürfnissen des Patienten. Hierzu gehören die Ermittlungen positiver wie negativer Erfahrungen [10]. Eine Untersuchung des AQUA-Instituts aus dem Jahre 2017 ergab hierbei, dass die 3 häufigsten Beschwerdegründe Organisation (z. B. lange Wartezeiten) mit 38,8 %, Zeitmangel des ärztlichen Personals oder Pflegepersonals mit 38,7 % und Kommunikation mit Ärzten mit 38,4 % darstellen [11]. Organisatorische und nichtmedizinische Faktoren haben demnach einen größeren Einfluss auf die Patientenzufriedenheit als die eigentliche medizinische Betreuung [12]. Die Aufnahme, Verarbeitung, Analyse und final ggf. Einleitung von Maßnahmen zur Verbesserung der Patientenzufriedenheit ist unter anderem wichtig, weil Patientinnen und Patienten im Gesundheitssystem auch Kunden sind, deren Zufriedenheit es nachhaltig zu sichern gilt. Dem Patienten muss durch eine patientenorientierte Aufnahme der Beschwerden deutlich gemacht werden, dass jede Beschwerde als Chance gesehen wird, die genannten Fehler nicht zu wiederholen. Dies setzt voraus, dass die Adressaten der Beschwerden sich substantiiert und kritisch reflektierend mit dem Inhalt auseinandersetzen und eine grundsätzliche Änderungsbereitschaft aufweisen. Nach patientenorientierter Sichtweise ist das Lob- und Beschwerdemanagement ein Indikator dafür, dass jeder Patient ernst genommen wird. Ein Patient, welcher sich ernst genommen fühlt in seinen Bedürfnissen, Erwartungen und partizipativen Entscheidungsfindungen, erhöht die Wahrscheinlichkeit einer verbesserten und erhöhten Patientenzufriedenheit [10]. Dies kann dazu beitragen, dass die Betroffenen dasselbe Krankenhaus erneut wählen und weiterempfehlen. Eine patientenseitig geäußerte Beschwerde kann als Signal interpretiert werden, dass der Patient wiederkommen möchte [12]. Gefühle eigener Enttäuschung oder Kränkung der Ärztinnen und Ärzte sollen dabei zurückstehen.

Es ist somit wichtig zu verstehen, dass jede Beschwerde wertvolle Informationen beinhaltet und gleichermaßen als Teil eines modernen Qualitätsmanagements etwaige Mängel aufzeigt. Dies sollte insbesondere vor dem Hintergrund der im Verhältnis zur Anzahl an Behandlungsvorgängen geringen Beschwerdezahlen Berücksichtigung finden. Im Mittel beträgt die Beschwerdequote (Anzahl Beschwerden × 100 / [ambulante Fälle + stationäre Fälle]) gerade einmal 0,72 % [13]. Denn für Patienten ist aufgrund des Abhängigkeitsverhältnisses zum Behandler häufig eine hohe Hemmschwelle zu überwinden, sodass diejenige/derjenige, der sie tatsächlich äußert, einen hohen Leidensdruck empfindet [12].

## Fazit für die Praxis

- Das Patientenrechtegesetz definiert die weitreichenden Patientenrechte.
- Patienten haben das Recht auf Herausgabe ihrer Behandlungsunterlagen.
- Lob- und Beschwerdemanagement ist ein wichtiger Teil des Qualitätsmanagements.
